# Supplementary material for: Inhibitory microcircuits for top-down plasticity of sensory representations
Source: Nat Commun. 2019 Nov 7;10:5055. doi: 10.1038/s41467-019-12972-2 (PMC6838080; doi:10.1038/s41467-019-12972-2)
Supplement: Supplementary file 1 — Supplementary Information [file 41467_2019_12972_MOESM1_ESM.pdf]

Inhibitory microcircuits for top-down plasticity of sensory representations

Katharina Anna Wilmes[1] and Claudia Clopath[1]

[1]Bioengineering Department, Imperial College London, SW72AZ London, UK

Supplementary Figures

To understand the sources of the synaptic changes, we analysed the spike timing of the different neuron types. (i) During the rewarded phase, VIPs were excited when the vertical bar was shown. As VIPs suppress SSTs, SSTs fired only briefly to the stimulus before they were silenced by VIPs. The lack of SST inhibition caused an increase in PV firing after the SSTs fired (Supplementary Figure 1b middle), such that spikes in SSTs preceded spikes in PVs during the vertical bar (Supplementary Figure 1d vertical SST - PV). As a consequence, connections from vertically tuned SSTs to PVs increased (Fig. 2b). (ii) During the refinement phase, the strong vertically tuned SST to PV connections led to increased inhibition of PVs. This disinhibited the PCs when a vertical bar was shown. As a result of this disinhibition, they increased their response towards the vertical bar and their likelihood to fire together. Additionally, vertically tuned PCs fired before the others as they received additional feedforward sensory input (Supplementary Figure 1c top and Supplementary Figure 1e vertical PC - angled PC). Therefore, vertically tuned PCs to other PCs connection strengthened. At the same time, the increased firing of PCs to the vertical bar increased PV firing just after vertically tuned SSTs responded. The vertically tuned SST-to-PV connections hence further strengthened (Fig. 3f).

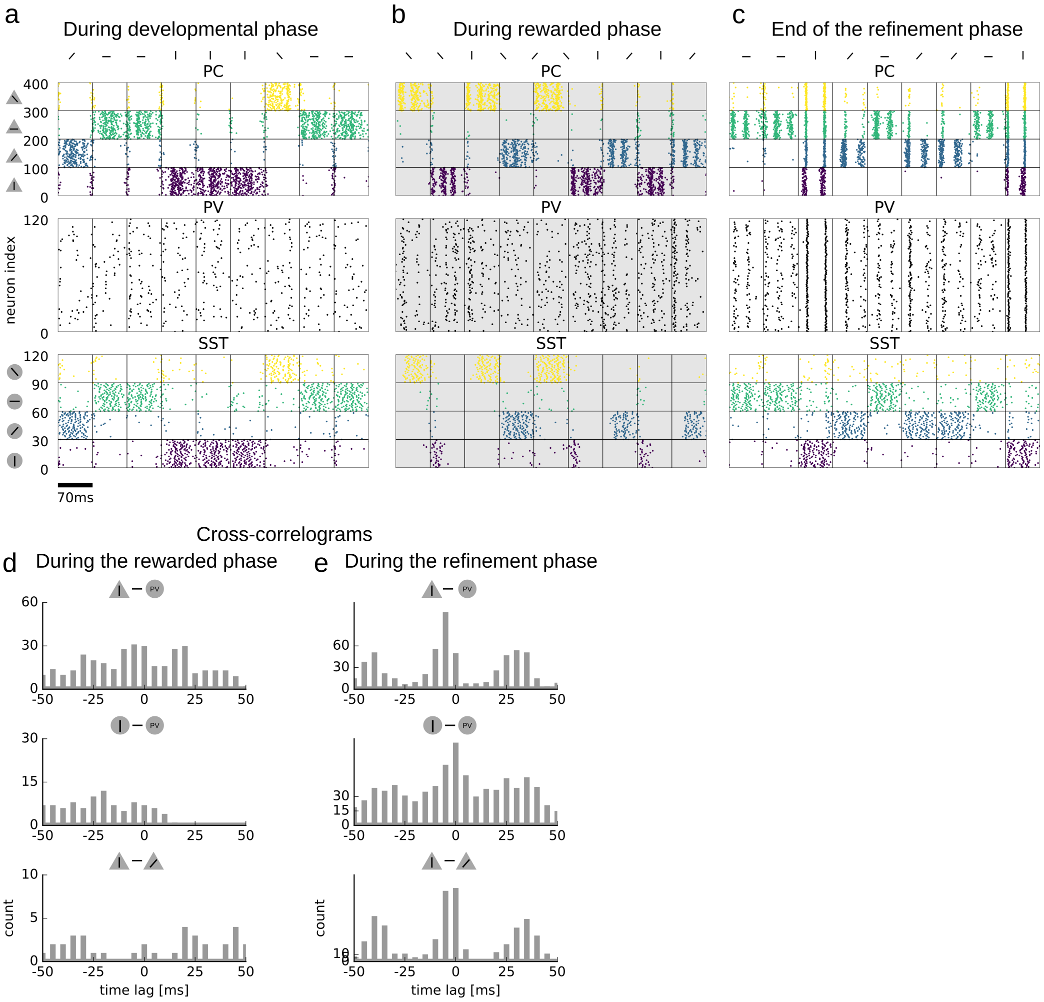

Supplementary Figure 1. Spiking information. a-c: Spike raster plots of PCs (top), PV interneurons (middle) and SSTs (bottom) during different stages of the simulation: at the beginning of the rewarded phase (a), during the rewarded phase (b, grey background), and in the end of the rewarded phase (c). Sensory inputs are presented for 50 ms, followed by a 20 ms stimulus gap. Changes of stimulus are indicated by vertical lines. d,e: Cross-correlograms for pairs of cells from different cell classes during (d) and after (e) the rewarded phase. Top: vertically tuned PC and PV. Middle: vertically tuned SST and PV. Bottom: vertically tuned PC and angled tuned PC.

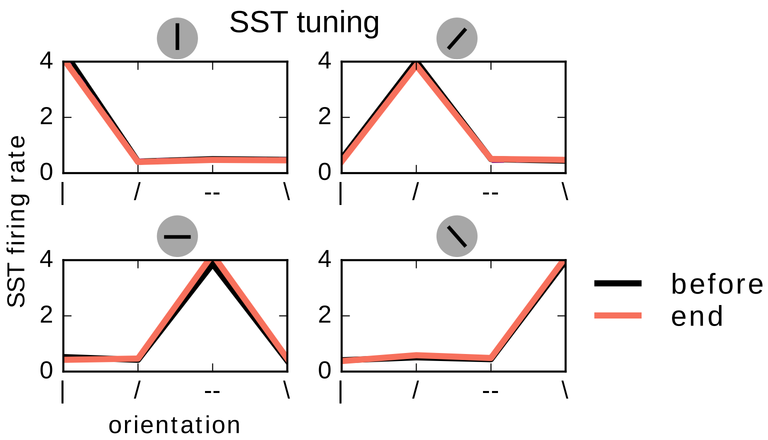

Supplementary Figure 2. SSTs slightly increase their response to the rewarded stimulus. Tuning of SST populations before the rewarded phase (black) and at the end of the refinement phase (red, number of spikes during 50 ms after stimulus onset averaged over all occurrences of that stimulus in 1 s of simulation). Tuning preference of each population is indicated above each panel.

The development of the excitatory structure depends on the selective disinhibition of PCs during the rewarded stimulus. The disinhibition of PCs during the rewarded phase in turn depends on the comparative strength of SST-PC and SST-PV-PC pathways. If the SST-PV-PC pathway is too weak, it fails to counteract the disinhibition via the VIP-SST-PC pathway. PV self-inhibition reduces the impact of the SST-PV-PC pathway, wheras PV gap junction coupling (c_{gap}) increases the impact of the SST-PV-PC pathway. We found that the degree to which excitatory structure developed depended on the strength of the PV gap junction coupling. The stronger the coupling, the less the excitatory structure develops during the rewarded phase (Supplementary Figure 3c) and the more it develops afterwards (Supplementary Figure 3d).

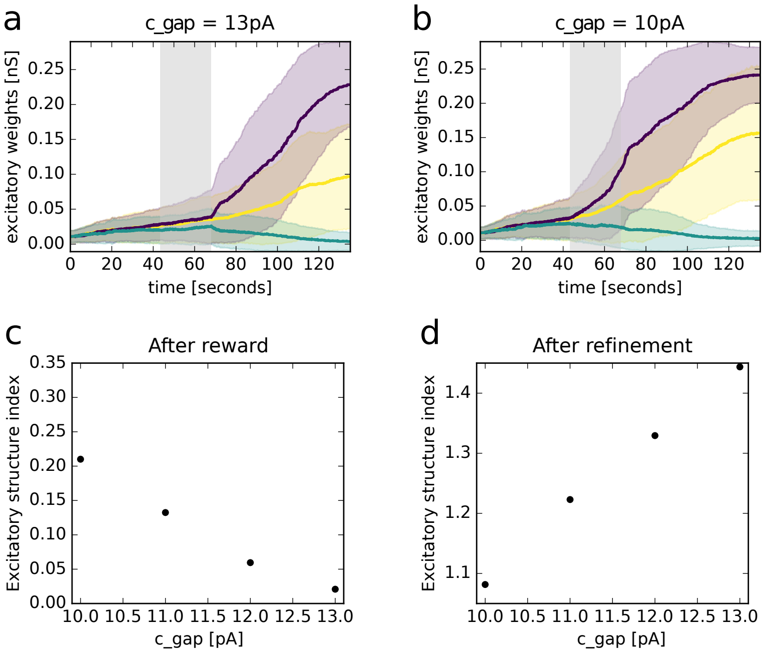

Supplementary Figure 3. Inhibitory and excitatory structure co-develop dependent on the disinhibition of PCs during the rewarded phase. a and b: Evolution of excitatory connections (mean and s.d.). Non-vertically tuned PCs to vertically tuned PCs (green), vertically tuned PCs to non-vertically tuned PCs (purple), non-vertically tuned PCs to non-vertically tuned PCs (yellow), for $c_{\text{gap}} = 10\,\text{pA}$ (a) and $13\,\text{pA}$ (b). c and d: Excitatory structure index (see methods) as a function of c_{gap} after the rewarded phase (c) and after the refinement phase (d).

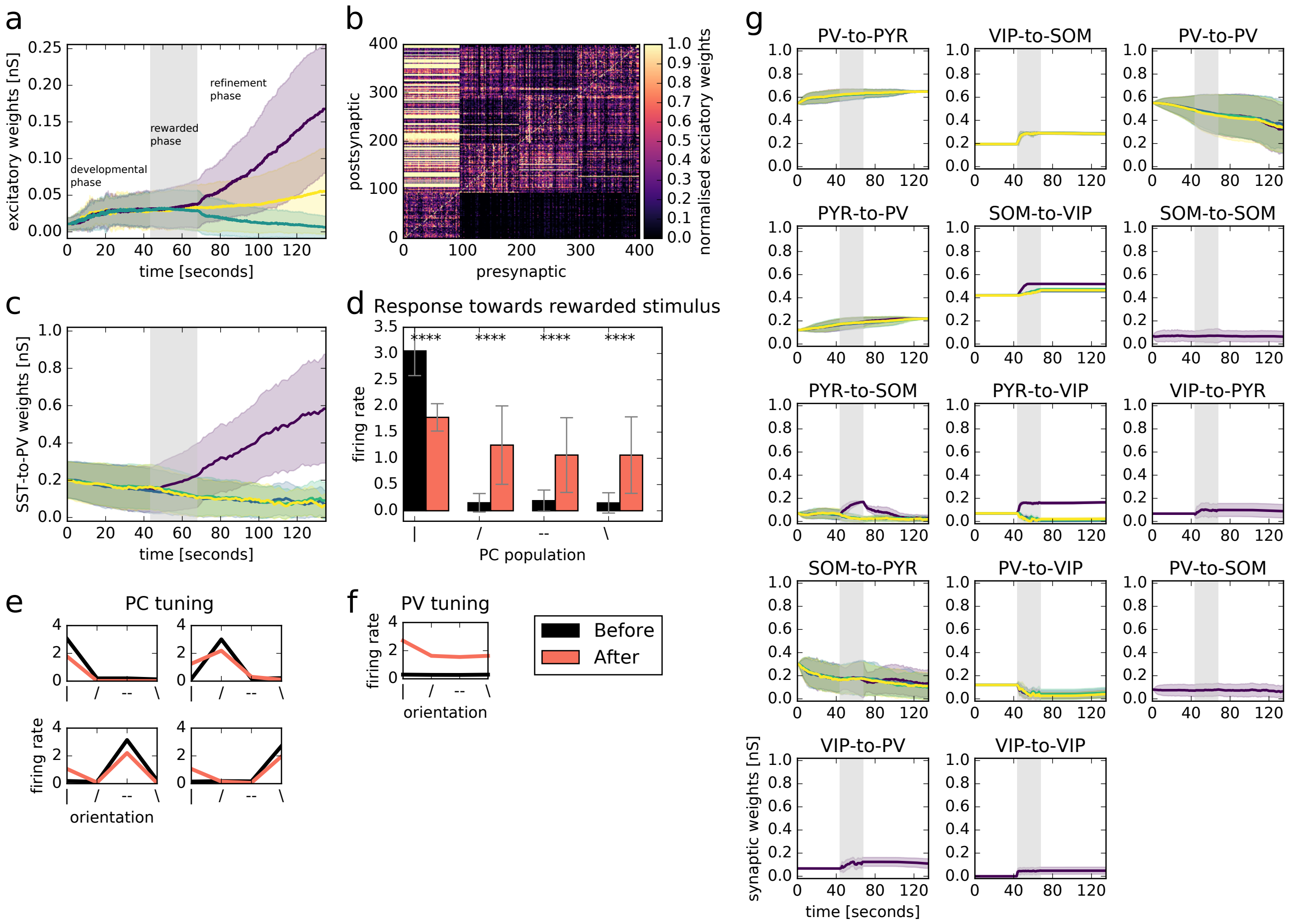

Supplementary Figure 4. Plasticity on all connections. Our model yields similar results if all connections are plastic. a: Evolution of excitatory connections (mean and s.d.). Non-vertically tuned PCs to vertically tuned PCs (green), vertically tuned PCs to non-vertically tuned PCs (purple), non-vertically tuned PCs to non-vertically tuned PCs (yellow). b: Recurrent excitatory weights at the end of the refinement phase. c: Evolution of the inhibitory SST-to-PV connections, grouped according to SST tuning preference (colours as in Fig. 2b, vertical in purple), shown are the mean and s.d. d: Mean and s.d. of the firing rates of cells in the four PC populations to the vertical stimulus before (black) and after learning (orange). **** indicates statistical significance with a p-value < 0.0001 from Student's t-test. e and f: Tuning of excitatory (e) and PV (f) populations before the rewarded phase (black) and at the end of the refinement phase (red) (number of spikes during 50 ms after stimulus onset averaged over all occurrences of that stimulus in 1 s of simulation). g-t: Evolution of connection strengths between different cell classes as indicated. Colour codes as before for the orientation tuning of the cell classes. Purple if neither class is orientation-tuned.

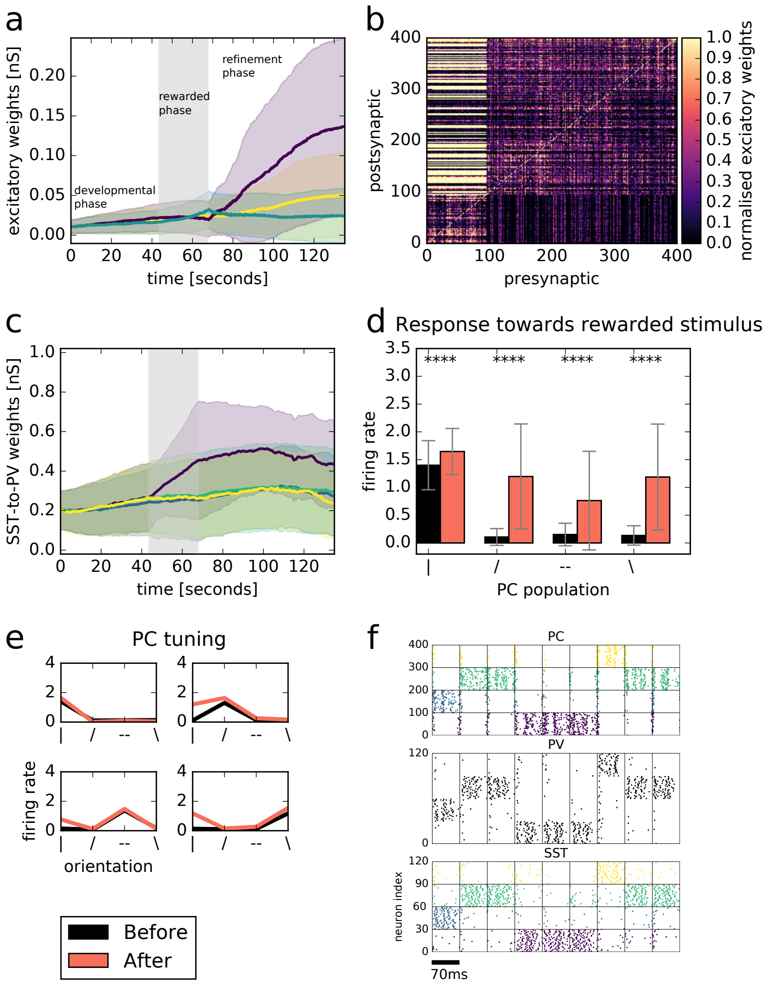

Supplementary Figure 5. The mechanism does not depend on the tuning of PV interneurons. a: Evolution of excitatory connections (mean and s.d.). Non-vertically tuned PCs to vertically tuned PCs (green), vertically tuned PCs to non-vertically tuned PCs (purple), non-vertically tuned PCs to non-vertically tuned PCs (yellow). b: Recurrent excitatory weights at the end of the refinement phase. c: Evolution of the inhibitory SST-to-PV connections, grouped according to SST tuning preference (colours as in Fig. 2b, vertical in purple), shown are the mean and s.d. d: Mean and s.d. of the firing rates of cells in the four PC populations to the vertical stimulus before (black) and after learning (orange). **** indicates statistical significance with a p-value < 0.0001 from Student's t-test. e: Tuning of excitatory populations before the rewarded phase (black) and at the end of the refinement phase (red) (number of spikes during 50 ms after stimulus onset averaged over all occurrences of that stimulus in 1 s of simulation). f: Spike raster plots of PCs (top), PV interneurons (middle) and SSTs (bottom) at the beginning of the simulation. Note that PVs are now sharply orientation-tuned.

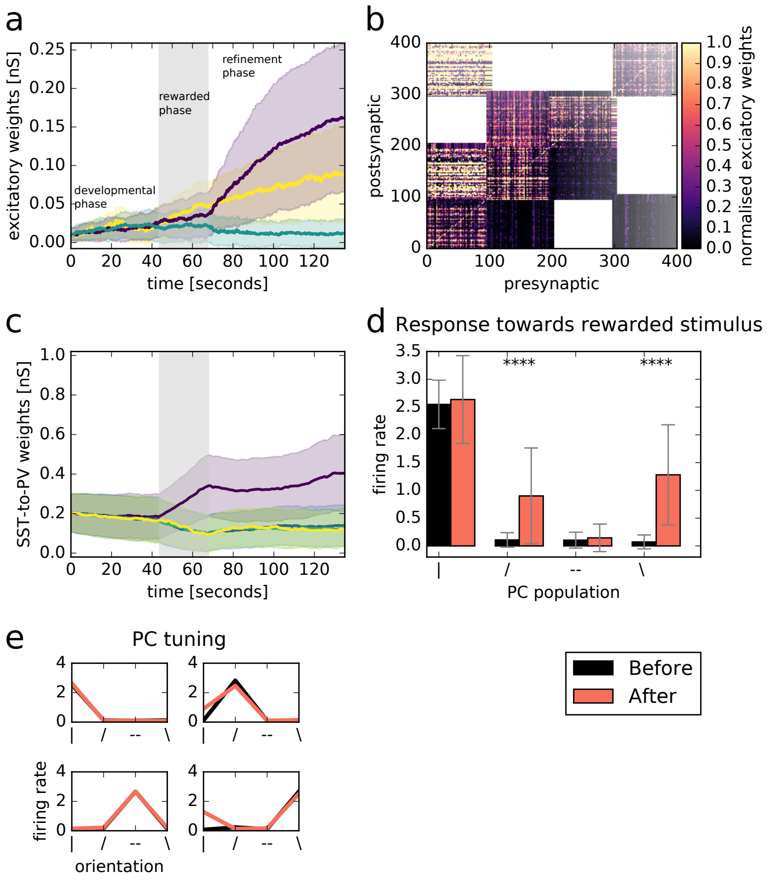

Supplementary Figure 6. Distance-dependent PC connectivity determines extent of the increase in stimulus representation. Model in which PCs are connected according to the cosine similarity of their preferred orientation, i.e. the connection probability of two cells is the cosine of the differences of their preferred angles. The horizontal population does not change its tuning as it is not connected to the vertical population. The angled populations are connected to the vertical population with a probability of 0.7. Thus those populations can increase their tuning to the vertical stimulus. a: Evolution of excitatory connections (mean and s.d.). Non-vertically tuned PCs to vertically tuned PCs (green), vertically tuned PCs to non-vertically tuned PCs (purple), non-vertically tuned PCs to non-vertically tuned PCs (yellow). b: Recurrent excitatory weights at the end of the refinement phase (white if there is no connection). c: Evolution of the inhibitory SST-to-PV connections, grouped according to SST tuning preference (colours as in Fig. 2b, vertical in purple), shown are the mean and s.d. d: Mean and s.d. of the firing rates of cells in the four PC populations to the vertical stimulus before (black) and after learning (orange). **** indicates statistical significance with a p-value < 0.0001 from Student's t-test. e: Tuning of excitatory populations before the rewarded phase (black) and at the end of the refinement phase (red) (number of spikes during 50 ms after stimulus onset averaged over all occurrences of that stimulus in 1 s of simulation).

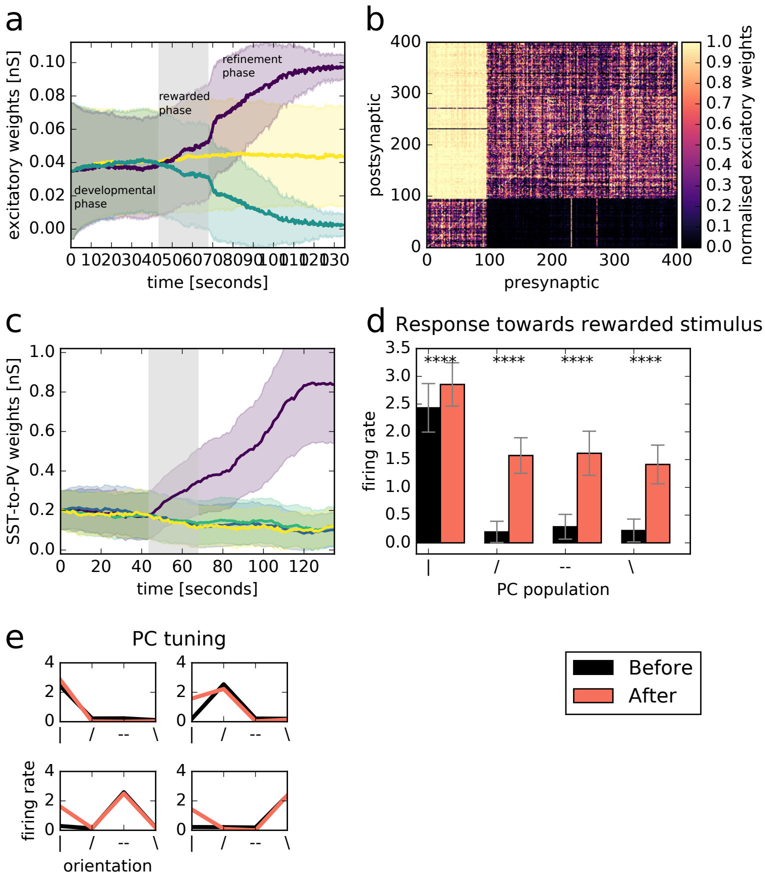

Supplementary Figure 7. Spiking model with stronger initial recurrent connectivity. a: Evolution of excitatory connections (initially sampled from $\mathcal{N}(.01, .1)$). Non-vertically tuned PCs to vertically tuned PCs (green), vertically tuned PCs to non-vertically tuned PCs (purple), non-vertically tuned PCs to non-vertically tuned PCs (yellow). b: Recurrent excitatory weights at the end of the refinement phase. c: Evolution of the inhibitory SST-to-PV connections, grouped according to SST tuning preference (colours as in Fig. 2b, vertical in purple), shown are the mean and s.d. d: Mean and s.d. of the firing rates of cells in the four PC populations to the vertical stimulus before (black) and after learning (orange). **** indicates statistical significance with a p-value < 0.0001 from Student's t-test. e: Tuning of excitatory populations before the rewarded phase (black) and at the end of the refinement phase (red, number of spikes during 50 ms after stimulus onset averaged over all occurrences of that stimulus in 1 s of simulation). Weights were bounded between 0 nS and 0.1 nS.

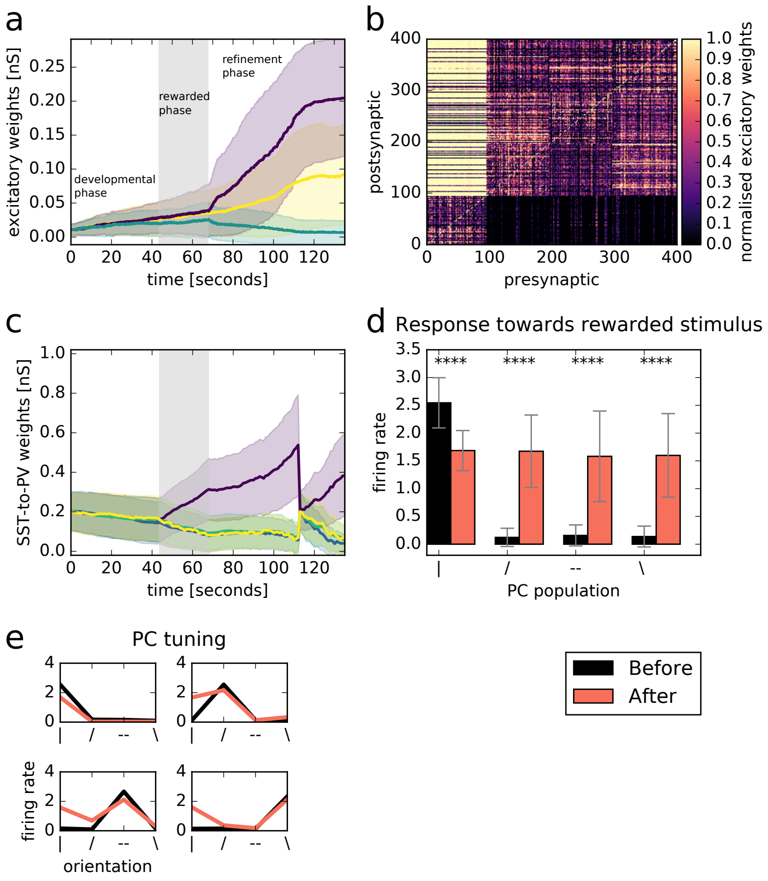

Supplementary Figure 8. Excitatory structure is stable without inhibitory structure. Simulation with same parameters as Fig. 2, but SST-to-PV connections were reset to their initial values at time 113.4 s. The excitatory structure remained stable. a: Evolution of excitatory connections (mean and s.d.). Non-vertically tuned PCs to vertically tuned PCs (green), vertically tuned PCs to non-vertically tuned PCs (purple), non-vertically tuned PCs to non-vertically tuned PCs (yellow). b: Recurrent excitatory weights at the end of the refinement phase. c: Evolution of the inhibitory SST-to-PV connections, grouped according to SST tuning preference (colours as in Fig. 2b, vertical in purple), shown are the mean and s.d. d: Mean and s.d. of the firing rates of cells in the four PC populations to the vertical stimulus before (black) and after learning (orange). **** indicates statistical significance with a p-value $<$ 0.0001 from Student's t-test. e: Tuning of excitatory populations before the rewarded phase (black) and at the end of the refinement phase (red) (number of spikes during 50 ms after stimulus onset averaged over all occurrences of that stimulus in 1 s of simulation).

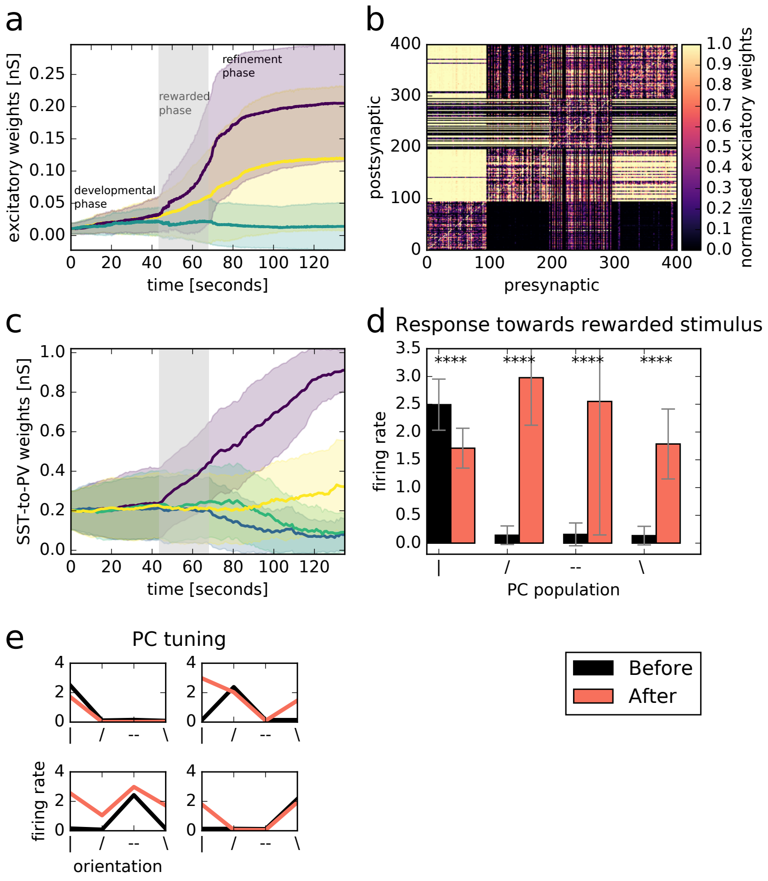

Supplementary Figure 9. Model in which PV gap junctions include a subthreshold coupling of w_{gap}=0.4 nS. a: Evolution of excitatory connections (mean and s.d.). Non-vertically tuned PCs to vertically tuned PCs (green), vertically tuned PCs to non-vertically tuned PCs (purple), non-vertically tuned PCs to non-vertically tuned PCs (yellow). b: Recurrent excitatory weights at the end of the refinement phase. c: Evolution of the inhibitory SST-to-PV connections, grouped according to SST tuning preference (colours as in Fig. 2b, vertical in purple), shown are the mean and s.d. d: Mean and s.d. of the firing rates of cells in the four PC populations to the vertical stimulus before (black) and after learning (orange). **** indicates statistical significance with a p-value < 0.0001 from Student's t-test. e: Tuning of excitatory populations before the rewarded phase (black) and at the end of the refinement phase (red) (number of spikes during 50 ms after stimulus onset averaged over all occurrences of that stimulus in 1 s of simulation).

Precise spike timing is not necessary: The two-stage model can be implemented in a rate coding scheme.

Although precise spike timing (1) and spike-timing based plasticity rules (2) have been reported in the primary visual cortex, it is unknown whether they play a role in enhancing stimulus representations. We hence investigated whether our two-stage model can also be implemented in a rate-based framework. Our network was in the inhibition-stabilized regime (ISN, Tsodyks et al. (3), recent experimental support: Moore et al. (4)) and the learning rule was a BCM type. In this regime, PC firing 'paradoxically' increases with increased PV activity. This is due to strong recurrent excitatory connections balanced by strong inhibition. Suppression of the inhibitory population in an ISN leads to an immediate increase in excitatory activity, which in turn drives the inhibitory population.

We implemented excitatory and inhibitory rate-based plasticity rules such that weight changes resembled those in the spiking implementation. (i) Excitatory plasticity favoured connections from high firing neurons to low firing neurons and depressed the reverse (see supplementary methods). The SST-to-PV plasticity potentiated synapses active while PVs fired above a threshold $\theta^{I}_{\mathrm{BCM}}$, reminiscent of the BCM learning rule (5).

During the rewarded phase, the SST-to-PV connectivity developed akin to the spiking model. In particular, connections from the vertically tuned SSTs to PVs strengthened, while the other connections weakened (Supplementary Figure 10b bottom, grey background). Additionally, the connection from the non-rewarded PCs to the rewarded vertically tuned PCs decreased (green line in Supplementary Figure 10b top).

During the refinement phase, PVs received more inhibition during the vertical stimulus (due to the strong connection from the vertically tuned SSTs). Nevertheless, the PV firing rate was higher during the vertical bar than during the horizontal stimulus (Supplementary Figure 10f around reward end). This reflects the ISN property of the network. Hence, the connection from the vertically tuned SST population continued to increase and remained stronger (Supplementary Figure 10b bottom) than the connection from the other SSTs. With the inhibitory structure in place, the vertically tuned PCs to horizontally tuned PCs connection strengthened (purple line in Supplementary Figure 10b top). Hence, the inhibitory structure remained stable and the excitatory structure developed during the refinement phase. The resulting excitatory connectivity resembles that in the spiking model (Supplementary Figure 10c).

As in the spiking model, both PCs and the PVs became more tuned to the rewarded stimulus (Supplementary Figure 10d and e). The excitatory populations simply increased their firing rate towards the rewarded stimulus (Supplementary Figure 10d). The PVs, however, increased their tuning by firing less towards the non-rewarded stimulus (Supplementary Figure 10e). The decrease in PV firing towards the non-rewarded stimulus resulted from an increased SST-to-PV connectivity from both SST populations.

Even though the spiking implementation yields similar results for weak and strong initial connectivity (Fig. 7), the rate implementation requires strong recurrent connectivity. To conclude, the proposed two-stage model can be realised even if only rate information is available. This happens provided that the network exhibits a counterintuitive increase of inhibitory firing rates, a property of inhibition-stabilised networks (3).

In the spiking model, inhibitory currents increase during the rewarded stimulus (Fig. 4d), whereas in the rate model inhibitory currents decrease during the non-rewarded stimulus (Supplementary Figure 10h). To delineate the spiking implementation from the rate coding implementation, the spiking model makes further predictions: (i) PVs fire more in synchrony (Supplementary Figure 1c middle), (ii) SSTs fire before PVs during the rewarded stimulus (Supplementary Figure 1d), (iii) PCs tuned to the rewarded stimulus fire before others during the rewarded stimulus after the task (Supplementary Figure 1e).

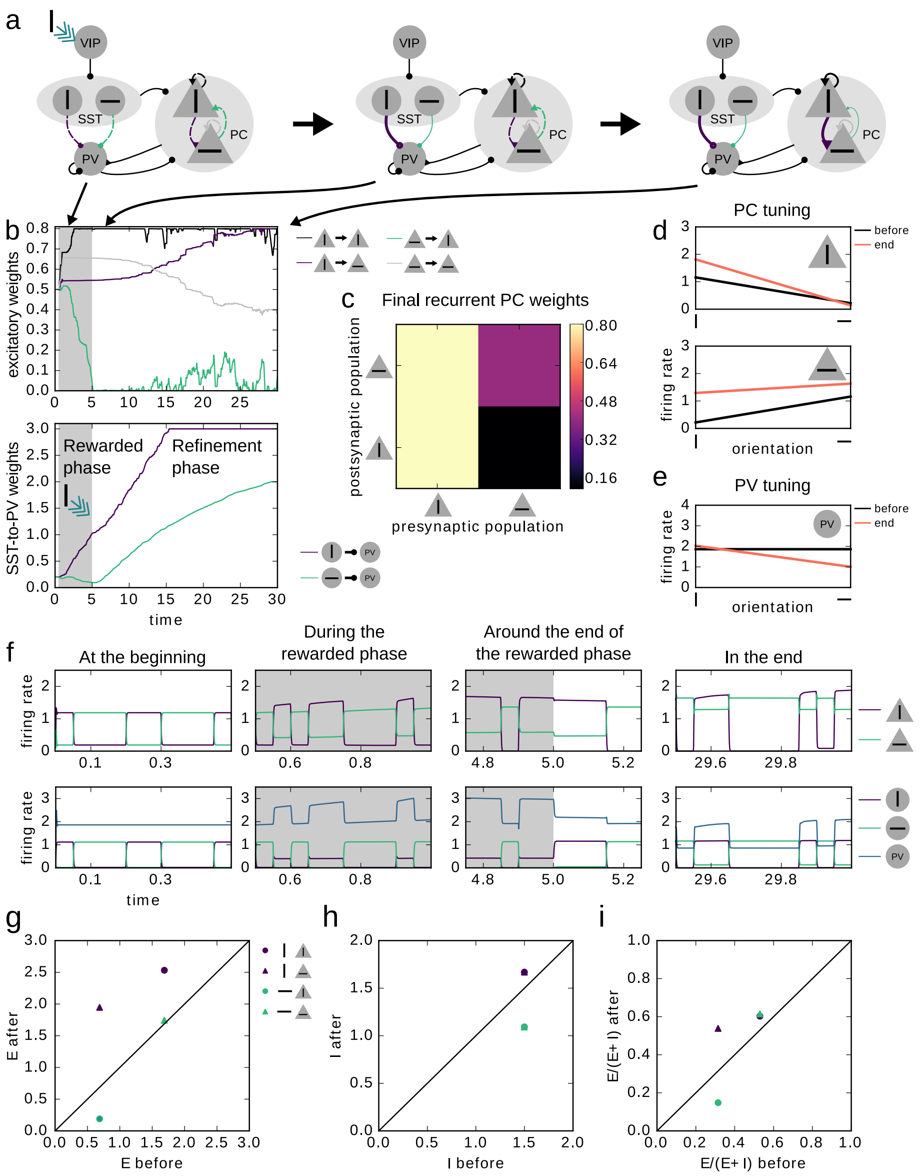

Supplementary Figure 10. The two-stage model of microcircuit plasticity can be realised in a rate coding scheme. a: Illustration of changes in inhibitory and excitatory connectivity. Snapshots of connectivity at the beginning (left), after the rewarded phase (middle) and at the end of the refinement phase (right). b: Top: Evolution of excitatory connections from the vertically tuned PC population to itself and to the horizontally tuned PCs, and from horizontally tuned PCs to horizontally tuned PCs (grey) and to vertically tuned PCs (green). Bottom: Evolution of connections from the vertically tuned SSTs to PVs (purple) and from the horizontally tuned SSTs to PV (green). Grey background highlights rewarded phase. C: Final excitatory connectivity matrix. d: Tuning curves of excitatory populations at the beginning and at the end of the simulation. e: Tuning curves of PV population at the same time points as in d. f: Firing rates of excitatory (top) and inhibitory (bottom) populations at the beginning of the simulations, during the rewarded phase, towards the end of the rewarded phase, and at the end of the simulation (from left to right). g-i: Excitatory (g), inhibitory (i), and E/I ratio (i) in excitatory populations (circles: vertically tuned, triangles: horizontally tuned) of rate model during the two different stimuli (purple: vertical stimulus, green: horizontal stimulus) before and after learning.

Supplementary Tables

Table 1: **Model assumptions and data**

	**Data**	**Assumptions**
Connectivity	I to E and I to I (6)	
	E to E and E to I (7; 8)<br>(9; 10)	
	Gap junctions between PVs (11)	Coupling strength
Inputs	Reward inputs to VIPs (12)	
	Stimulus inputs to PCs (13),<br>PVs (13; 14),<br>SSTs (15)	Input strength
Tuning	orientation-tuned PCs (16; 17)	orientation-tuned
	orientation-tuned SSTs (16; 17)	orientation-tuned
	broadly tuned PVs (16; 17),<br>(7; 18) but see (19)	either tuned or untuned
Plasticity	Excitatory STDP (20)	
	anti-Hebbian PV-to-PC (21)	
	SST-to-PV (no cell type-specific data)<br>VIP-SST and PC-SST (no cell type-specific data)<br>other connections (no cell type-specific data)	STDP<br>anti-Hebbian STDP<br>STDP

Table 2: **Postdictions and Predictions**

**Postdictions**	**References**
Similarly tuned PCs form clusters.	(22; 23)
The stimulus representation increases.	(24–26)
PCs and PVs increase their response to the rewarded stimulus.	(24; 27)
**Predictions**	
PVs initially decrease, then increase their response.	
SSTs slightly increase their response.	
PC and PV interactions increase, and fire more in synchrony.	
Both E and I currents in PCs increase during the rewarded stimulus.	
The E/I ratio in PCs increases during the rewarded stimulus.	

Supplementary Methods

The excitatory structure index is defined as the difference between the mean weight from vertically tuned PCs to all other neurons and the mean weight from PCs not coding for vertical bars to vertically tuned PCs, normalised by the maximum weight:

$$\Big(\frac{1}{P(N-P)}\sum_i\sum_j W_{ij} - \frac{1}{(N-P)(N-2P)}\sum_k\sum_l W_{kl}\Big)/\max W \tag{1}$$

with j indexing vertically tuned PCs ($1 < j < P$), and i indexing other PCs ($i > P$). If $k \in K$ then $l \notin K$, and $k, l > P$, where P is the number of PCs in one population, and N is the total number of PCs.

Connections from PVs to PCs (21), PCs to SSTs, and VIPs to SSTs were plastic according to anti-Hebbian STDP, wheras all other connections followed classical STDP (see main methods). The learning rate η was 0.1 for plasticity at SST-to-VIP synapses. For anti-Hebbian STDP, weight changes follow the following update rule:

$$\Delta w = \begin{cases} A_- \exp(\frac{\Delta t}{\tau_+}) & \text{if } \Delta t < 0 \\ -A_+ \exp(-\frac{\Delta t}{\tau_-}) & \text{if } \Delta t \geq 0 \end{cases} \tag{2}$$

The parameters were the same as for classical STDP (see main methods). Unless otherwise stated, synaptic weights had a relative upper bound of 0.1 nS above their initial value.

In the simulations with tuned PVs (Supplementary Figure 5), PVs receive the same layer 4 input as PCs and SSTs. The 120 PV neurons are split up into four groups with 30 neurons each receiving input from one of four tuned layer 4 inputs. The conductance of synapses from L4 to PVs was 0.2 nS.

In the rate model, the activity of a population of neurons i is described by their firing rate r_i, which evolves over time according to:

$$\tau_i \frac{dr_i}{dt} = -r_i + \phi(I_i^{\text{ext}} + \sum_j W_{ij} r_j) \tag{3}$$

where $i, j \in \{\text{E1, E2, P, S1, S2, V}\}$ and τ_i is the time constant of population i. $I_i^{\text{ext}} = I_{\text{baseline}} + I_{\text{stimulus}} + I_{\text{neuromodulation}}$, $\sum_j W_{ij} r_j$ is recurrent input. $\phi(x)$ is the activation function given by

$$\phi(x) = \begin{cases} 0 & \text{if } x < 0 \\ (r_{\text{max}} - r_0) tanh(x/(r_{\text{max}} - r_0)) & \text{if } x \geq 0 \end{cases} \tag{4}$$

Each of the PCs and SSTs receive an input current I_{stimulus} upon presentation of their preferred stimulus. PCs also receive a constant baseline current input I_{baseline}. The VIPs receive a neuromodulatory current $I_{\text{neuromodulation}}$ when the rewarded stimulus (vertical bar) is present. r_0 and r_{max} denote the minimum and maximum firing rate, respectively.

In the rate model, the initial connectivity is taken such that the network is in the ISN regime.

$$W = \begin{pmatrix} W_{\text{EE}} & W_{\text{EP}} & W_{\text{ES}} & W_{\text{EV}} \\ W_{\text{PE}} & W_{\text{PP}} & W_{\text{PS}} & W_{\text{PV}} \\ W_{\text{SE}} & W_{\text{SP}} & W_{\text{SS}} & W_{\text{SV}} \\ W_{\text{VE}} & W_{\text{VP}} & W_{\text{VS}} & W_{\text{VV}} \end{pmatrix} = \begin{pmatrix} .5 & .5 & .5 & 0 \\ 2.2 & .5 & .2 & 0 \\ .1 & 0 & 0 & .3 \\ 0 & 0 & 0 & 0 \end{pmatrix} \tag{5}$$

Plasticity in the rate model was modelled as follows: For excitatory connections, we used a BCM-like rule (5), where the sign of synaptic change depends on whether the activity of the postsynaptic neuron exceeds a threshold. The rule is Hebbian as the weight change depends on the product of pre- and post-synaptic activity. The weight change follows

$$\frac{dw^{\text{E}}}{dt} = \alpha^{\text{E}} r_{\text{post}}(r_{\text{pre}} - \theta_{\text{BCM}}) r_{\text{pre}} \tag{6}$$

where α^{E} is the excitatory learning rate, r_{pre} is the presynaptic firing rate, r_{post} the postsynaptic firing rate, and $\theta_{\text{BCM}}^{\text{E}}$ is the sliding threshold. The threshold is sliding and changes according to:

$$\tau_{\text{BCM}}^{\text{E}} \frac{d\theta_{\text{BCM}}}{dt} = -\theta_{\text{BCM}}^{\text{E}} + r_{\text{post}} \frac{r_{\text{post}}}{\theta_{\text{target}}} \tag{7}$$

where θ_{target} is the target firing rate and $\tau^{\text{E}}_{\text{BCM}}$ the time constant.

For inhibitory connections, we used:

$$\frac{dw^{\text{I}}}{dt} = \alpha^{\text{I}} r_{\text{pre}}(r_{\text{post}} - \theta^{\text{I}}_{\text{BCM}}) r^2_{\text{post}} \quad (8)$$

where α^{I} is the learning rate, r_{pre} is the presynaptic firing rate, r_{post} the postsynaptic firing rate, and $\theta^{\text{I}}_{\text{BCM}}$ is the inhibitory sliding threshold. It changes according to:

$$\tau^{\text{I}}_{\text{BCM}} \frac{d\theta^{\text{I}}_{\text{BCM}}}{dt} = -\theta^{\text{I}}_{\text{BCM}} + r_{\text{post}} \frac{r_{\text{post}}}{\theta^{\text{I}}_{\text{target}}} \quad (9)$$

where θ_{target} is the target firing rate and $\tau^{\text{I}}_{\text{BCM}}$ the time constant.

Table 3: **Parameters of the rate model.**

Parameter	Value	Parameter	Value
τ_i	1.0	α^{E}	5.0e-4
r_0	1.0	$\tau^{\text{E}}_{\text{BCM}}$	1/1.0e-2
r_{max}	20.0	θ_{target}	1.5
I_{stimulus}	1.0	$W^{\text{I}}{}_{\text{maxsum}}$	5.0
I_{baseline}	1.0	$W^{\text{I}}{}_{\text{max}}$	3.0
$I_{\text{neuromodulation}}$	2.5	α^{I}	1.0e-4
W_{max}	.8	$\tau^{\text{I}}_{\text{BCM}}$	1/1.0e-3
W_{maxsum}	1.2	$\theta^{i}_{\text{target}}$	3.2

All rate model simulations were done with a time step of 0.1 [arb. unit]. First, the model was simulated for 500 [arb. unit] without plasticity to measure the tuning properties. Then plasticity was switched on, and the rewarded phase started. The rewarded phase ended at time 5000 [arb. unit]. The refinement phase ended at time 30000 [arb. unit].

Supplementary References

References

[1] Tiesinga, P., Fellous, J.-M. & Sejnowski, T. J. Regulation of spike timing in visual cortical circuits. *Nature Reviews Neuroscience* **9**, 97 (2008). Review Article.

[2] Sjöström, P. J., Turrigiano, G. G. & Nelson, S. B. Rate, timing, and cooperativity jointly determine cortical synaptic plasticity. *Neuron* **32**, 1149–1164 (2001).

[3] Tsodyks, M., Skaggs, W. E., Sejnowski, T. J. & Mcnaughton, B. L. Paradoxical effects of inhibitory interneurons. *J. Neurosci.* **17**, 4382–4388 (1997).

[4] Moore, A. K., Weible, A. P., Balmer, T. S., Trussell, L. O. & Wehr, M. Rapid rebalancing of excitation and inhibition by cortical circuitry. *Neuron* **97**, 1341 – 1355.e6 (2018).

[5] Bienenstock, E., Cooper, L. & Munro, P. Theory for the development of neuron selectivity: orientation specificity and binocular interaction in visual cortex. *Journal of Neuroscience* **2**, 32–48 (1982).

[6] Pfeffer, C. K., Xue, M., He, M., Huang, Z. J. & Scanziani, M. Inhibition of inhibition in visual cortex: The logic of connections between molecularly distinct interneurons. *Nature neuroscience* **16**, 1068–1076 (2013).

[7] Hofer, S. B. *et al.* Differential connectivity and response dynamics of excitatory and inhibitory neurons in visual cortex. *Nature Neuroscience* **14**, 1045 EP – (2011).

[8] Jiang, X. *et al.* Principles of connectivity among morphologically defined cell types in adult neocortex. *Science* **350** (2015).

[9] Pala, A. & Petersen, C. C. In vivo measurement of cell-type-specific synaptic connectivity and synaptic transmission in layer 2/3 mouse barrel cortex. *Neuron* **85**, 68 – 75 (2015).

[10] Jouhanneau, J.-S., Kremkow, J. & Poulet, J. F. A. Single synaptic inputs drive high-precision action potentials in parvalbumin expressing gaba-ergic cortical neurons in vivo. *Nature Communications* **9**, 1540 (2018).

[11] Galarreta, M. & Hestrin, S. A network of fast-spiking cells in the neocortex connected by electrical synapses. *Nature* **402**, 72–75 (1999).

[12] Pi, H.-J. *et al.* Cortical interneurons that specialize in disinhibitory control. *Nature* **503**, 521–524 (2013).

[13] Adesnik, H., Bruns, W., Taniguchi, H., Huang, Z. J. & Scanziani, M. A neural circuit for spatial summation in visual cortex. *Nature* **490**, 226 EP – (2012).

[14] Kloc, M. & Maffei, A. Target-specific properties of thalamocortical synapses onto layer 4 of mouse primary visual cortex. *Journal of Neuroscience* **34**, 15455–15465 (2014).

[15] Pakan, J. M., Francioni, V. & Rochefort, N. L. Action and learning shape the activity of neuronal circuits in the visual cortex. *Current Opinion in Neurobiology* **52**, 88–97 (2018). Systems Neuroscience.

[16] Ma, W.-p. *et al.* Visual representations by cortical somatostatin inhibitory neurons—selective but with weak and delayed responses. *Journal of Neuroscience* **30**, 14371–14379 (2010).

[17] Cottam, J. C. H., Smith, S. L. & Häusser, M. Target-specific effects of somatostatin-expressing interneurons on neocortical visual processing. *Journal of Neuroscience* **33**, 19567–19578 (2013).

[18] Atallah, B. V., Bruns, W., Carandini, M. & Scanziani, M. Parvalbumin-expressing interneurons linearly transform cortical responses to visual stimuli. *Neuron* **73**, 159–170 (2012).

[19] Runyan, C. A. *et al.* Response features of parvalbumin-expressing interneurons suggest precise roles for subtypes of inhibition in visual cortex. *Neuron* **67**, 847–857 (2010).

[20] Bi, G. & Poo, M. Synaptic modifications in cultured hippocampal neurons: Dependence on spike timing, synaptic strength, and postsynaptic cell type. *Journal of Neuroscience* **18**, 10464–10472 (1998).

[21] Vickers, E. D. *et al.* Parvalbumin-interneuron output synapses show spike-timing-dependent plasticity that contributes to auditory map remodeling. *Neuron* **99**, 720 – 735.e6 (2018).

[22] Clopath, C., Büsing, L., Vasilaki, E. & Gerstner, W. Connectivity reflects coding: a model of voltage-based STDP with homeostasis. *Nature Neuroscience* **13**, 344 (2010). Article.

[23] Ko, H. *et al.* The emergence of functional microcircuits in visual cortex. *Nature* **496**, 96 (2013).

[24] Poort, J. *et al.* Learning enhances sensory and multiple non-sensory representations in primary visual cortex. *Neuron* **86**, 1478 – 1490 (2015).

[25] Goltstein, P. M., Coffey, E. B. J., Roelfsema, P. R. & Pennartz, C. M. A. In vivo two-photon Ca^{2+} imaging reveals selective reward effects on stimulus-specific assemblies in mouse visual cortex. *Journal of Neuroscience* **33**, 11540–11555 (2013).

[26] Goltstein, P. M., Meijer, G. T. & Pennartz, C. M. Conditioning sharpens the spatial representation of rewarded stimuli in mouse primary visual cortex. *eLife* **7**, e37683 (2018). URL `https://doi.org/10.7554/eLife.37683`.

[27] Khan, A. G. *et al.* Distinct learning-induced changes in stimulus selectivity and interactions of GABAergic interneuron classes in visual cortex. *Nature Neuroscience* **21**, 851–859 (2018).
